# Small Molecules in the Venom of the Scorpion *Hormurus waigiensis*

**DOI:** 10.3390/biomedicines8080259

**Published:** 2020-07-31

**Authors:** Edward R. J. Evans, Lachlan McIntyre, Tobin D. Northfield, Norelle L. Daly, David T. Wilson

**Affiliations:** 1 Centre for Molecular Therapeutics, AITHM, James Cook University, Cairns, QLD 4878, Australia; edwardrobertjonathan.evans@my.jcu.edu.au (E.R.J.E.); norelle.daly@jcu.edu.au (N.L.D.); 2 Independent Researcher, P.O. Box 78, Bamaga, QLD 4876, Australia; lach.mcintyre@gmail.com; 3 Department of Entomology, Tree Fruit Research and Extension Center, Washington State University, Wenatchee, WA 98801, USA; TNORTHFIELD@WSU.EDU

**Keywords:** venom, scorpion, adenosine, purine, nucleoside, nucleotide, citric acid, glutamic acid, aspartic acid

## Abstract

Despite scorpion stings posing a significant public health issue in particular regions of the world, certain aspects of scorpion venom chemistry remain poorly described. Although there has been extensive research into the identity and activity of scorpion venom peptides, non-peptide small molecules present in the venom have received comparatively little attention. Small molecules can have important functions within venoms; for example, in some spider species the main toxic components of the venom are acylpolyamines. Other molecules can have auxiliary effects that facilitate envenomation, such as purines with hypotensive properties utilised by snakes. In this study, we investigated some non-peptide small molecule constituents of *Hormurus waigiensis* venom using LC/MS, reversed-phase HPLC, and NMR spectroscopy. We identified adenosine, adenosine monophosphate (AMP), and citric acid within the venom, with low quantities of the amino acids glutamic acid and aspartic acid also being present. Purine nucleosides such as adenosine play important auxiliary functions in snake venoms when injected alongside other venom toxins, and they may have a similar role within *H. waigiensis* venom. Further research on these and other small molecules in scorpion venoms may elucidate their roles in prey capture and predator defence, and gaining a greater understanding of how scorpion venom components act in combination could allow for the development of improved first aid.

## 1. Introduction

Scorpion envenomation poses a significant public health issue in certain areas of the world, particularly in northern Saharan Africa, South and East Africa, the Near- and Middle-East, southern India, Mexico, Brazil, and within the Amazonian basin [1]. Although the effects of scorpion venom can be severe when injected by a scorpion, individual venom components can have a wide range of positive applications in medicine when administered in a controlled way [2]. Past research into scorpion venoms has primarily focused on peptide constituents, as these molecules display the greatest diversity within the venom, are often responsible for the greatest toxic effects, and have the largest potential for development of therapeutics or bioinsecticides [3,4,5,6]. However, scorpions possess complex venom containing a mixture of proteins, peptides, small molecules, and salts [7]. Whilst non-peptide small molecules are often reported to be present within scorpion venom, and despite being known to play important roles in the venom of different taxa [8,9,10], the identities and functions of small molecules in scorpion venom remain poorly described. Identifying the small molecules present in scorpion venoms may allow a greater understanding of how scorpion venoms function, have evolved, and may help improve treatment of stings. Additionally, certain venom-derived small molecules may have applications as therapeutics [11]. At present, only a very small number of non-peptide small molecules (<1 kDa) have been confidently characterised from scorpion venoms, including an alkaloid described from *Megacormus gertschi* [12], two 1,4-benzoquinone derivatives from *Diplocentrus melici* [13], adenosine from *Heterometrus laoticus* [14,15], and citric acid from *Centruroides sculpturatus* [16]. Other published material identified the presence of spermidine in the venom of *Palamneus phipsoni* (*Heterometrus phipsoni*) [17,18] and 5-hydroxytryptamine (serotonin) in *Leiurus quinquestriatus* and *Buthotus minax* (*Hottentotta minax*) [17,19]; however, no definitive data were collected, and therefore re-examination of these venoms with modern analytical techniques would help to confirm their presence. Furthermore, free amino acids, nucleotides, lipids, amines, heterocyclic compounds, and inorganic salts are reportedly present in scorpion venoms [4,20,21], but their specific molecular compositions and functions remain generally unknown.

Venomous organisms commonly possess low-molecular weight non-peptide molecules within their venom [9,10]. The identities and functions of the low-molecular weight non-peptide molecules found in different organisms may provide insights into those that may be found in scorpions. Spiders are one of the most closely related groups of venomous organisms to scorpions, and are also one of the most widely studied groups of venomous animals. Different spider species possess a large range of small molecules within their venom, including acylpolyamines and polyamines [11,22,23,24,25,26]; alkaloids [27,28,29]; nucleosides, nucleotides, and analogues [30,31,32,33,34,35]; and free-amino acids [33,36], alongside other molecules that have been subject to less intensive study [37,38,39]. Given that scorpions use their venom against similar predators and prey to spiders, the small molecules they possess may be similar to those found in spiders.

Small molecules can act directly as toxins within venom, have facilitatory roles that increase the overall toxicity or effectiveness of the venom, or alternatively have functional roles linked to the maintenance of toxins within the venom gland [8,40,41,42]. For example, polyamines in the venom of spiders in the genus *Nephila* are sufficiently toxic towards insects to directly aid in the incapacitation of prey [42]. Alternatively, nigriventrine from the Brazilian armed spider (*Phoneutria nigriventer*) can induce convulsions in mammals, and therefore help the spider to defend against predators [40]. Whilst such molecules directly contribute to venom toxicity when targeting prey or potential predators, some small-molecule venom constituents do not induce a toxic effect when injected into a target organism, but can still play an important role within venom by acting in conjunction with toxins to facilitate envenomation or incapacitation of the target [8,43]. For example, purine nucleosides such as adenosine, guanosine, and inosine are present within a wide range of elapid and viperine snake venoms, and whilst these molecules generally have very low toxicity and are naturally present within the target organism, injection of high concentrations alongside other toxins can improve the overall effectiveness of the venom by facilitating envenomation and incapacitation [8,43]. Aird [8] explains that snake venom has three primary modes of action against prey—immobilisation by hypotension, immobilisation by paralysis, and digestion—and that the suite of small molecules injected have key roles in all three functions. As the body of research investigating the small molecule constituents of snake venoms is expanding, there is growing understanding that non-toxic small molecules can play important auxiliary roles within venoms, and future research should aim to elucidate the complex interactions that occur during envenomation [10]. In addition to these functions as toxins or facilitators of toxins, small molecules can have functions within the venom gland associated with maintenance and production of toxins. For example, citrate, which can inhibit the action of venom proteins within the gland, may be in the venom to prevent self-harm [16,41,44]. As scorpion venoms contain a suite of uncharacterised small molecules, it is possible that some may induce toxicity directly, have auxiliary effects that increase overall venom toxicity, or have functions associated with the production and storage of toxins. 

In this study, we investigated some of the small molecule compositions of Australian rainforest scorpion (*Hormurus waigiensis*) venom using reversed-phase high-performance liquid chromatography (RP-HPLC), liquid chromatography/mass spectrometry (LC/MS), and nuclear magnetic resonance (NMR) spectroscopy. *H. waigiensis* is a burrowing scorpion that is widely distributed across Southeast Asia, the Pacific, New Guinea, and Australia [45]. Within Australia, it is found in tropical and sub-tropical forests along the eastern coast from northern New South Wales to Cape York, with reports of populations also present in the Northern Territory and Western Australia [46]. Stings from this species are considered mild, with the venom causing moderate pain and swelling [47]. Presently just one toxin, φ-liotoxin-Lw1a (φ-LITX-Lw1a), has been characterised from *H. waigiensis* [48,49]. The toxin φ-LITX-Lw1a was the first scorpion toxin reported to adopt a disulfide-directed β-hairpin (DDH) structure and may provide a missing link explaining how the three-disulfide inhibitor cystine knot (ICK) motif has evolved in scorpions [48,49]. The remaining constituents of *H. waigiensis* venom have not been characterised. Scorpion venom research has historically been skewed towards medically significant species, all of which belong to the family Buthidae [50]. *H. waigiensis* is a member of the family Hormuridae, which has been subject to far fewer investigations by toxinologists. Given the structural novelty of φ-LITX-Lw1a, *H. waigiensis* venom may contain other molecules with significant structural differences to those present in more thoroughly studied Buthid venoms.

## 2. Materials and Methods

### 2.1. Scorpion Collection

Scorpions were collected from rainforest sites in the vicinity of Kuranda (QLD, Australia), and kept on the premises of Minibeast Wildlife, Kuranda. The scorpions were housed in plastic food containers with substrate and a piece of bark, and kept at ambient temperature. Prior to milking, individuals had been maintained in captivity for less than one year.

### 2.2. Venom Extraction and Purification

Venom samples were collected from *H. waigiensis* using a square-wave stimulator (Arthur H. T. Thomas Co. Scientific Apparatus, Philadelphia, PA, USA) to electrostimulate the venom gland at 25V DC, 0.5 ms pulse duration, at a frequency of 1 pulse/sec. The samples were pooled into 20 µL of MilliQ water and stored at −20 °C. Pooled samples collected on two separate dates were further pooled prior to analysis. A further five scorpions (two male and three female) were milked and the samples were stored separately in 30 µL of MilliQ water for LC/MS analysis.

Crude pooled venom was fractionated by reversed-phase HPLC (RP-HPLC) using a Phenomenex Jupiter^®^ C_18_ column (250 × 10 mm, 10 µm, 100 Å; Phenomenex, Torrence, CA, USA). Fractionation of the venom components was achieved using a linear gradient of two mobile phases: H_2_O/0.05% trifluoroacetic acid (TFA; Auspep, Tullamarine, VIC, Australia) (solvent A) and 90% acetonitrile (ACN; Sigma-Aldrich, St. Louis, MO, USA)/H_2_O/0.045% TFA (solvent B). Separation used a gradient of 0–60% solvent B in 120 min, 60–90% solvent B in 5 min, 90% solvent B for 10 min, and 90–0% solvent B in 5 min, at a flow rate of 3 mL/min. The venom component elution was monitored at 214 nm and 280 nm, and 0.5 min fractions were collected.

### 2.3. Liquid Chromatography/Mass Spectrometry (LC/MS)

Five individual scorpion venom samples and serial dilutions of adenosine and adenosine monophosphate (AMP) standards were analysed by liquid chromatography/mass spectrometry (LC/MS) using a Shimadzu LCMS-2020 mass spectrometer coupled to a Shimadzu Prominence HPLC system (Shimadzu, Kyoto, Japan) to allow quantitation of these molecules within the venom. A small amount of pooled venom was also analysed by LC/MS to observe the composition and determine the molecular weights of the molecules present. Then, 8 µL of the individually stored venom samples were injected in triplicate, 5 µL of the adenosine and AMP serial dilutions were injected in triplicate, and 10 µL of pooled venom (3 µL in 7 µL MilliQ water) was injected. The samples were injected via an autosampler (Shimadzu SIL-20AC_HT_) onto a reversed-phase high-performance liquid chromatography (HPLC) column (Phenomonex Aeris 3.6 µm PEPTIDE XB-C18 100 Å; Phenomenex, Torrence, CA, USA) at 30 °C. Solvent delivery (solvent A: 0.1% formic acid (FA; Sigma-Aldrich, St. Louis, MO, USA)/water; solvent B: 90% acetonitrile (ACN; OPTIMA LCMS grade, Thermo Fisher Scientific, Scoresby, VIC, Australia)/0.09% formic acid/water) was via Shimadzu LC-20AD pumps at a flow rate of 0.250 mL/min. Samples were eluted with a 1% gradient (0–60% solvent B, 60 min; 60–90% solvent B, 5 min; 90% solvent B, 5 min; 90-–0% solvent B, 5 min; 0% solvent B, 10 min), and the UV absorbance was observed at 214 nm and 280 nm on a Shimadzu SPD-20A detector. Mass spectra were collected in positive ion mode over a scan range of *m*/*z* 130–2000 and negative mode with a scan range of *m*/*z* 200–2000, with a detector voltage of 1.15 kV, nebulizing gas flow of 1.5 L/min, and drying gas flow of 3.0 L/min. Data were collected and analysed using the Shimadzu LabSolutions v5.96 software (Shimadzu, Kyoto, Japan). The 280 nm peak areas for the peaks containing adenosine and AMP, both in the individual venom samples and serial dilutions, were exported and analysed further in R version 3.6.1 [51]. The peak areas for standard samples and venom samples run in triplicate were averaged prior to further analysis. A linear model was fit to the serial dilution data to produce a standard curve, which was used to predict the quantity of adenosine and AMP in the 8 µL injection of each venom sample. These quantities were then divided by eight and multiplied by thirty to provide an estimate of the quantity contained within the whole venom samples. As the crude venoms were suspended in 30 μL of water, the total volume would be slightly greater than 30 μL; therefore, we calculated a slight underestimate of the quantity of adenosine and AMP contained within the venom.

### 2.4. Mass Spectrometry and NMR Analysis

NMR spectra were recorded at 290 K on a Bruker Avance III 600 MHz spectrometer (Bruker, Billerica, MA, USA) equipped with a cryoprobe. Samples were dissolved in 90% H_2_O/10% D_2_O (*v*/*v*) (100 μM). D_2_O (99.9%) was obtained from Cambridge Isotope Laboratories, Woburn, MA, USA, for ^1^H NMR measurements. Spectra were referenced to the water signal. Two-dimensional spectra included TOCSY, NOESY, DQF-COSY, HSQC, HMBC, and HSQC-TOCSY. TOCSY and NOESY mixing times of 80 ms and 500 ms, respectively, were used. Spectra were analysed using Topspin v3.6.1(Bruker, Billerica, MA, USA).

High-resolution mass spectrometry (MS) was performed using a SCIEX TOF/TOF™ 5800 MALDI (SCIEX, Framingham, MA, USA) and a SCIEX TripleTOF 6600 (SCIEX, Framingham, MA, USA) mass spectrometers. Matrix-assisted laser desorption ionisation mass spectrometry (MALDI-MS) samples were spotted on 384-well stainless steel target plates using 0.5 μL of sample and 0.5 μL of either α–cyano-4-hydroxycinnamic acid (CHCA; Sigma-Aldrich, St. Louis, MO, USA) matrix (7.5 mg/mL in 50% ACN/0.1% TFA) or a 2,5-dihydroxybenzoic acid (DHB; Sigma-Aldrich, St. Louis, MO, USA) matrix (10 mg/mL in 50% ethanol/0.1% TFA). Calibration was performed before spectra collection for each sample using Calibration Mix Solution 2 (SCIEX, Framingham, MA, USA). Spectra were acquired in reflector positive ion mode from *m*/*z* 200 to 400, and averaged over 2000 laser shots. Tandem-MS (MS/MS) was performed in 1 kV positive ion mode with collision-induced dissociation (CID) and averaged over 2000 shots. Samples were manually infused to the SCIEX TripleTOF 6600 mass spectrometer equipped with a DuoSpray Ion Source using the syringe pump and a 1 mL 4.61 mm i.d. Hamilton syringe (Reno, NV, USA) at a flow rate of 5 μL/min. Data acquisition was performed with Analyst^®^ TF 1.7.1 software (SCIEX, Framingham, MA, USA). Product ion mass spectra were collected in positive ion mode with an accumulation time of 500 ms and a mass range of *m*/*z* 100 to 400. The source temperature was 470 °C, the curtain gas was set to 45 psi, the ion source gas 1 and 2 were set to 40 and 50 psi respectively, and the ion-spray voltage floating set to 4.9 kV. MS/MS data were collected on manually selected product ions under the same conditions with an accumulation time of 250 ms and a mass range of *m*/*z* 20 to 350. 

## 3. Results

### 3.1. Venom Collection and Fractionation

**Figure 1:**
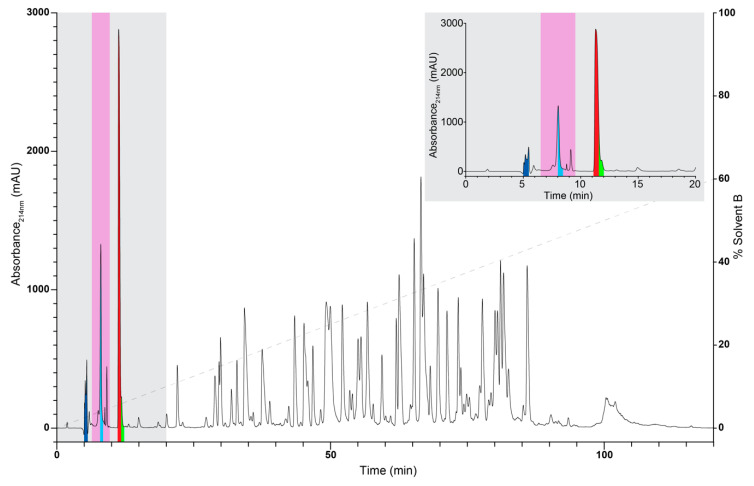
Reversed-phase high performance liquid chromatography (RP-HPLC) chromatogram of pooled crude *H. waigiensis* venom (Phenomenex Jupiter^®^ C_18_ column; 250 × 10 mm, 10 µm, 100 Å; 3 mL/min flow rate; solvent A H_2_O/0.05% TFA, solvent B 90% ACN/H_2_O/0.045% TFA; 0–60% solvent B in 120 min, 60–90% solvent B in 5 min, 90% solvent B for 10 min, and 90–0% solvent B in 5 min; absorbance at 214 nm). The inset shows an expanded view of the highlighted area in the full chromatogram. The fractions of interest and corresponding peaks in the chromatogram are highlighted in dark blue (glutamic acid and aspartic acid), red (adenosine), and light blue (adenosine monophosphate), respectively. The pink section shows the fractions containing citric acid. The fraction highlighted in green is likely to be inosine. The dashed line shows the solvent B gradient.

Crude venom pooled from ~15 venom extractions of *H. waigiensis* scorpions was fractionated using RP-HPLC with a C_18_ semi-preparative RP-HPLC column, and 30 s fractions were collected (see Figure 1). A small sample of the pooled venom (3 µL) was also subjected to liquid chromatography/mass spectrometry (LC/MS) analysis, which provided a venom mass profile and identified the presence of a number of small molecules in early eluting peaks. 

### 3.2. NMR Analysis of RP-HPLC Fractions

**Figure 2:**
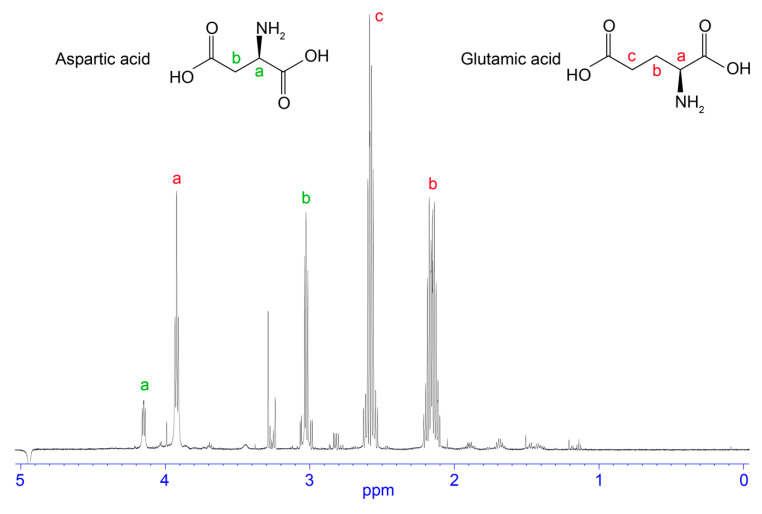
Chemical structure and 1D NMR spectrum of a fraction from *H. waigiensis* venom containing aspartic acid and glutamic acid. The assignments were derived based on two-dimensional NMR spectra and one-dimensional NMR spectra of glutamic acid and aspartic acid standards.

**Figure 3:**
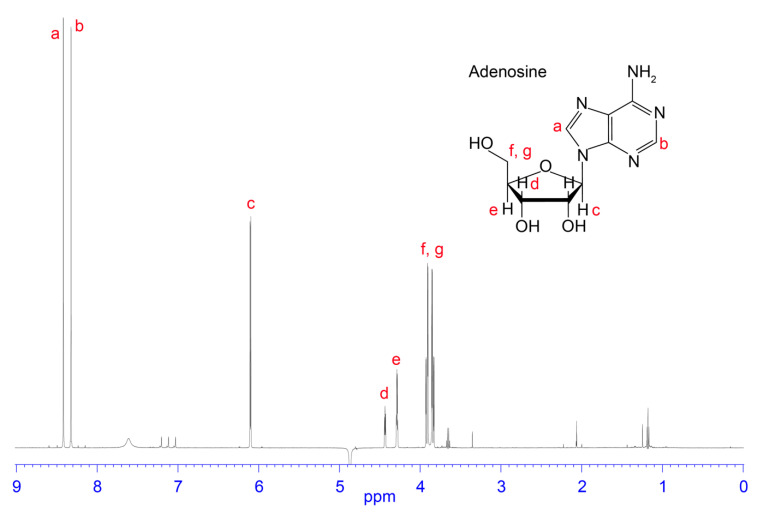
Chemical structure and 1D NMR spectrum of a fraction from *H. waigiensis* venom containing adenosine. The assignments were derived based on one-dimensional NMR spectra of an adenosine standard.

**Figure 4:**
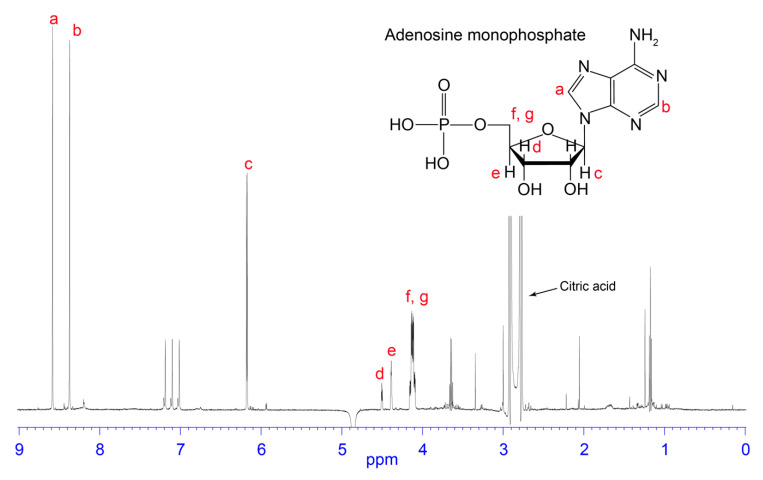
Chemical structure and 1D NMR spectrum of a fraction from *H. waigiensis* venom containing adenosine monophosphate. The assignments were derived based on one-dimensional NMR spectra of an adenosine monophosphate standard. The presence of citric acid is also highlighted.

**Figure 5:**
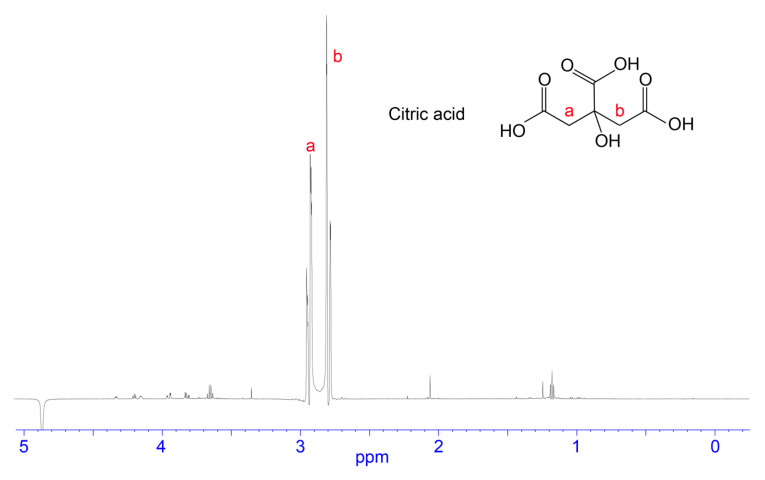
Chemical structure and 1D NMR spectrum of a fraction from *H. waigiensis* venom containing citric acid. The assignments were derived based on one-dimensional NMR spectra of a citric acid standard.

Fractions collected from 5.5 to 23 min were analysed by one-dimensional ^1^H NMR spectroscopy to observe the presence of small molecules, typified by sharp peaks and an absence of or minimal peaks in the amide region (see Figure 2, Figure 3, Figure 4 and Figure 5). Fractions corresponding to the RP-HPLC peaks highlighted in dark blue and red in Figure 1 appeared to be relatively clean and produced good signal-to-noise ratios in the ^1^H NMR spectra. Therefore, these fractions were selected for further analysis by two-dimensional NMR spectroscopy and the structures were elucidated based on correlations observed in the HMBC, HSQC, and COSY spectra. The dark blue peak highlighted in Figure 1 represented 1.28% of the total peak area within the 214 nm RP-HPLC chromatogram and was shown to contain glutamic acid and aspartic acid (see Figure 2). The red peak (Figure 1) represented 7.68% of the total peak area within the 214 nm RP-HPLC chromatogram and contained adenosine (see Figure 3). AMP (see Figure 4) was present within the light blue peak, which represented 2.42% of the total peak area of the RP-HPLC chromatogram (Figure 1). Fractions collected in the pink coloured region (Figure 1) contained citric acid, although as it does not have an absorbance profile at 214 nm nor 280 nm, it was not observed in the RP-HPLC chromatogram and was characterised based on NMR data (Figure 5). The green peak representing 0.33% of the total peak area of the 214 nm RP-HPLC chromatogram (Figure 1) contained a molecule with a mass 1 Da greater than adenosine, suggesting it may be inosine; however, we were unable to confirm its identity due to its low abundance and coelution with adenosine. A molecule was also observed with the mass of inosine monophosphate (IMP); however, low abundance and coelution with AMP on the RP-HPLC system prevented us from confirming its identity. Full 1D NMR spectra of RP-HPLC fractions and standards labelled with peak integration values used for molecular characterisation are contained within the Supplementary Materials.

### 3.3. High-Resolution Mass Spectrometry

High-resolution mass spectrometry data collected using a SCIEX TripleTOF 6600 (SCIEX, Framingham, MA, USA) and SCIEX TOF/TOF™ 5800 MALDI (SCIEX, Framingham, MA, USA) were consistent with and confirmed the elucidated structures. For the dark blue peak fraction, [M + H]^+^ ions were observed at *m*/*z* 148.0350 and *m*/*z* 134.0187, confirming the presence of glutamic acid (147.053158 Da) and aspartic acid (133.037508 Da). For the red peak fraction, a [M + H]^+^ ion was observed at *m*/*z* 268.0783, confirming the presence of adenosine (267.096741 Da). Using a precursor ion of *m*/*z* 268.1, the red peak fraction showed a strong ion at *m*/*z* 136.0827 corresponding to adenine following fragmentation and cleavage of the ribose. For the light blue peak fraction, an [M + H]^+^ ion was observed at *m*/*z* 348.0649, confirming the presence of AMP (347.063084 Da). Using a precursor ion of *m*/*z* 348.06 on this fraction showed an ion at *m*/*z* 136.1006, which also corresponded to adenine following fragmentation and cleavage of the ribose.

### 3.4. Adenosine Quantitation by LC/MS

Five adult scorpions were milked individually to estimate the quantities of adenosine and AMP contained within the total venom by comparing the LC/MS 280 nm absorbance peak areas against those of a serial dilution of standards run under identical chemical conditions. All five scorpion venoms contained adenosine. Quantities of approximately 4.462 μg and 2.448 μg were present within the two male venoms; and 4.555 μg, 2.016 μg, and 5.808 μg were present within the female venoms. The abundance of AMP displayed greater variation between individuals and was absent from two of the female scorpions at detectable levels. The estimated quantity of AMP within the whole venoms of the two males were 0.682 μg and 2.778 μg, while the single female venom contained 0.318 μg. It is important to note that these values are slight underestimates of the true quantity contained within the venom gland. By comparing the volumes of venom collected in the pipette tip against set volumes of water, we estimated that each scorpion produced between 1.5 µL and 3.5 µL during milking, and therefore an average milking contained roughly 2.5 µL of venom. This indicates that the values calculated from 30 µL of the sample are slight underestimates of the true total, but this variation is small in comparison to the large differences observed between individuals.

## 4. Discussion

Adenosine, AMP, citric acid, glutamic acid, and aspartic acid were all found to be present within the venom of *H. waigiensis*. Molecules were also observed with masses corresponding to inosine and IMP; however, we were unable to confirm their identity due to low abundances and coelution with adenosine and AMP, respectively. 

### 4.1. Adenosine

Adenosine has been previously identified within the venom of the scorpion *Heterometrus laoticus* [14,15], and is a known constituent of other arthropod venoms, including those belonging to the spiders *Haplopelma lividum* and *Latrodectus menavodi* [31,52], the wasp *Cyphononyx dorsalis* [53], and the ant *Pseudomyrmex triplarinus* [54]. Inosine was also found to be present within the venom of *L. menavodi* [31] and other spider species, including *Parawixia bistriata* [32], *Cyriopagopus hainanus* (*Selenocosmia huwena*) [55], *Loxosceles reclusa* [56], and six more species [57]. Purine nucleosides such as adenosine and inosine are common constituents of elapid and viperine snake venoms and are thought to play important accessory roles in helping snakes envenomate and incapacitate their prey [8,10,43,58]. Adenosine has a wide range of physiological effects in mammalian targets, including inducing vasodilation, causing increased vascular permeability, increasing blood coagulation time, and inhibiting neurotransmitter release [8,14]. The effects of inosine are not so well understood, but it is also known to increase vascular permeability and induce hypotension through selective binding with mast cell A_3_ receptors [59], as well as having vasodilatory properties ([60]; reviewed in [8]). Aird [8] provides an overview of the large body of literature studying the physiological effects of adenosine and inosine in mammalian targets, and summarises how these properties may translate to a functional role within a venom. For example, by inducing vasodilation and increasing vascular permeability venomous organisms may improve their systemic envenomation of the target [8,10,43,58]. 

Whilst the effects of adenosine and inosine in mammalian targets are relatively well understood, scorpions do not solely utilise their venom to defend against mammals [61]. Unlike elapid and viperid snakes, which frequently target vertebrates, scorpions predominantly hunt invertebrates, although in certain environments they are known to take small vertebrates such as blind snakes [62]. Whilst Aird [8] discusses the role of adenosine and inosine in snake venoms that target mammalian prey, it is unclear how closely this translates to scorpions aiming to incapacitate invertebrate prey. Purinoreceptors evolved at an early date, and therefore structural similarities exist between the receptors that vertebrates and invertebrates possess [63]. It is, therefore, possible that adenosine will interact with invertebrate and vertebrate purinoreceptors in similar ways [8,63], but morphological differences between them may lead to different overall effects. For example, insects and mammals have distinctly different circulatory systems, and therefore the hypotensive effects induced by adenosine in mammals may not directly translate to an insect model. It is, however, likely that other effects of adenosine, such as reducing the release of neurotransmitters, may be more closely paralleled between insects and mammals [43]. IMP induces delayed paralysis when injected into termites, but it remains untested whether inosine would have the same effect [32]. Because *H. waigiensis* scorpions generally attack invertebrate prey but have to defend themselves against vertebrates, their venom contains components effective specifically against vertebrates and invertebrates, as well as components against both [64]. It is currently unclear whether adenosine facilitates the envenomation and incapacitation of invertebrate prey when injected alongside other venom components, or whether it is more heavily involved in defence against vertebrates. It could also function within the venom to aid capture of small vertebrate prey in environments with low invertebrate prey abundance [62].

### 4.2. AMP

In addition to adenosine, we identified AMP within the venom of *H. waigiensis*. Similar to adenosine, AMP has hypotensive properties, and therefore could act in a similar way to facilitate envenomation in mammalian aggressors [43,65]. Aird [43] found that only a small number of snake venoms contained AMP compared with adenosine, but stated that the presence of purine monophosphates within venoms is unsurprising due to the hypotensive effects they share with free purines. We are unaware of previous reports of AMP, adenosine diphosphate (ADP), or adenosine triphosphate (ATP) from scorpion venoms; however, they are present within different tarantula venoms, including those belonging to *H. lividum*, *Lasiodora* sp., *Aphonopelma* sp., and *Aphonopelma hentzi* (*Dugesiella hentzi* and *Eurypelma californicum*) [30,33,52,66,67]. Unlike adenosine, which binds to P1 receptors, ADP and ATP bind to P2 receptors [68], with evidence suggesting that they also have auxiliary roles alongside toxins within venom [30,66]. As with adenosine, ADP and AMP possess hypotensive properties and may fulfil a similar auxiliary function within the venom [8,66]. On the other hand, ATP is less vasodilatory and can lead to hypertension [8,65], and therefore may function differently within the venom. However, Chan, Geren, Howell, and Odell [30] showed that ATP works synergistically with the specific toxins to increase toxicity towards mice. It is possible that adenosine and AMP may act in a similar way to increase the overall toxicity of scorpion venom. To test this, future work could perform a bioassay injecting adenosine and AMP alongside other venom toxins, looking for synergistic or auxiliary effects. Of the five scorpions we tested, the whole venoms contained between 2.016 μg and 5.808 μg of adenosine. Intravenous injection of much lower quantities (0.1 μg) in mice induced hypotension [65], while topically applied 10^−5^ M adenosine solution increased vascular permeability in hamster cheek pouches [69]. This suggests that it is likely that the quantity of adenosine contained within the venom is large enough to elicit such effects at the site of injection.

### 4.3. Citric Acid/Citrate

Citric acid or citrate is a common constituent of venoms, present within spider, snake, bee, wasp, ant, and scorpion venoms [16,38,41]. Its presence within spider venoms is particularly well documented, having been recorded from at least 48 species belonging to 16 families [38]. Despite the common occurrence of citrate within venoms, its role is not fully understood, and it may serve multiple functions. One proposed function of venom citrate is the inhibition of toxins within the venom gland, thereby preventing self-harm [44]. As citrate can act as a chelator and form strong complexes with divalent metal ions such as Ca^2+^, Mg^2+^, and Zn^2+^, it can inhibit venom proteins that are dependent on these metal ions [44]. Citrate, therefore, inhibits venom proteins such as calcium-ion-dependant phospholipase A_2_ (PLA_2_) neurotoxins and myotoxins, as well as zinc-ion-dependant venom metalloprotease haemorrhagic toxins [38,41]. For example, it has been demonstrated experimentally that honey bee (*Apis mellifera*) venom PLA_2_ is at least partially inhibited by citrate [16], and that the citrate concentration present within the fer-de-lance snake (*Bothrops asper*) venom is high enough to likely completely inhibit PLA_2_ and 5′-nucleotidase activity, whilst partially inhibiting (75%) phosphodiesterase activity [44]. Scorpion venoms can contain both PLA_2_ proteins [70] and metalloproteases [71] within their venom; therefore, citrate may act as an inhibitor of these toxins within the venom gland. One study found that dried Arizona bark scorpion (*Centruroides sculpturatus*) venom contained 7.77 ± 1.2% citrate, but the extent that citrate is present in the venoms of different scorpion species remains unknown [16]. It is, therefore, important to test more scorpion venoms for the presence of citrate, particularly those well known to contain PLA_2_ or metalloproteases, to identify if this is its primary function within scorpion venom. Other proposed functions of citric acid within the venom gland include having direct antimicrobial effects or enhancing the effects of antimicrobial molecules [38,72]. Furthermore, it has been suggested that in spiders citric acid may be present to counter cationic peptides and acylpolyamines within the venom gland [38]. At the time of writing no acylpolyamines have been characterised from scorpion venoms, but cationic peptides are present [73,74,75]. It is likely that citrate has multiple functions within the venom gland of scorpions, but additionally it may have a role once injected into the target organism. An example of this has been demonstrated with the cardiotoxin A3 (CTX A3) from the Taiwan cobra (*Naja atra*), where heparin-sulphate-mediated cell retention of CTX A3 is citrate dependant [76]. However, it is currently unknown if citrate acts in conjunction with any scorpion toxins within an envenomated organism.

### 4.4. Free Amino Acids

Free amino acids have been widely reported from snake venoms [10,77] and spider venoms [33,36,78], but to our knowledge their possible functions within the venoms remain unknown. We found aspartic acid and glutamic acid to be present within *H. waigiensis* venom, but these molecules were only present in low abundance (Figure 1). The fraction containing glutamic acid and aspartic acid contained other small molecules at lower abundance, indicated by the presence of extra peaks within the NMR spectra (Figure 2), but we were unable to characterise these molecules. A cocktail of amino acids is naturally found in body fluids and can be released from degrading cells, and these molecules may not have a function contributing to the toxicity of the venom. However, if this was the case we might expect to see a more diverse range of amino acids present within the venom. It is possible that other amino acids are present within the venom of *H. waigiensis* and we were only able to identify aspartic acid and glutamic acid as we were able to attain the cleanest NMR spectra for these molecules, but the presence of these same amino acids in spider venoms suggests they may have some specific function. In tarantulas, glutamic acid has been found in the venom of *H. lividum*, and in two separate studies both aspartic acid and glutamic acid were identified from *A. hentzi* [33,36]. Furthermore, NMR screening has shown that aspartic acid is found within the venom of the spider *Pisaura mirabilis*, while glutamic acid is a common component of many different araneomorph spider venoms [34]. Glutamic acid, or the conjugate base glutamate, is an important neurotransmitter in the central nervous system [79], and injection in certain parts of the body will likely disrupt the nervous system. More work is required to explain the presence of these molecules in venom.

### 4.5. Conclusions

Whilst scorpion venoms have been subject to intensive study, research has been heavily skewed to focus on peptide venom constituents. Members of the family Buthidae have also been subject to more intensive study than other scorpion families, as Buthidae contains all medically significant species [50]. *H. waigiensis* belongs to the family Hormuridae [80], which has received comparatively little attention from researchers. It may be that members of this family and other neglected families also utilise small molecules within their venom arsenals. To our knowledge, adenosine has previously been reported from just one other scorpion species, *H. laoticus*, which belongs to the family Scorpionidae [15]. Scorpions show great toxin diversity between species, and the presence of adenosine in two scorpion species belonging to different families suggests that it may be more commonly utilised by other species. Furthermore, to our knowledge this study provides the first direct evidence of nucleotides (AMP) and specific amino acids within a scorpion venom. Our investigation highlights that the small molecules contained within *H. waigiensis* venom are similar to those found in other closely and distantly related venomous organisms, suggesting that such molecules may have important functions in the venoms of a wide range of organisms. Future work should aim to investigate the extent that small molecules are utilised by different scorpion species, paying particular attention to understudied families. Characterising the suite of small molecules within scorpion venoms will help us gain a greater understanding of the biochemical mechanisms involved in scorpion envenomation. This understanding may allow for the development of improved and optimised treatment of scorpion envenomation, or allow facilitation or modulation of the action of developed scorpion-venom-derived therapeutics and bioinsecticides. Furthermore, the abundance and diversity of small-molecule venom constituents in different species of scorpions remains largely unstudied, and uncharacterised bioactive molecules could have potential applications in pharmacology.

## Supplementary Materials

The following are available online at https://www.mdpi.com/2227-9059/8/8/259/s1.
