# Supplementary material for: Small Molecules in the Venom of the Scorpion Hormurus waigiensis
Source: Biomedicines. 2020 Jul 31;8(8):259. doi: 10.3390/biomedicines8080259 (PMC7459668; doi:10.3390/biomedicines8080259)
Supplement: Supplementary file 1 [file biomedicines-08-00259-s001.pdf]

Small molecules in the venom of the scorpion *Hormurus waigiensis*

Edward R. J. Evans [1], Lachlan McIntyre [2], Tobin D. Northfield [3], Norelle L. Daly [1] and David T. Wilson [1,*]

[1] Centre for Molecular Therapeutics, AITHM, James Cook University, Cairns, QLD, 4878, Australia
[2] PO Box 78, Bamaga, QLD, 4876, Australia
[3] Tree Fruit Research and Extension Center, Department of Entomology, Washington State University, Wenatchee, WA, USA

edwardrobertjonathan.evans@my.jcu.edu.au (E.R.J.E.); lach.mcintyre@gmail.com (L.M.); tnorthfield@wsu.edu (T.D.N.); norelle.daly@jcu.edu.au (N.D.); david.wilson4@jcu.edu.au (D.T.W.)

* Correspondence: david.wilson4@jcu.edu.au; Tel.: +61-7-4232-1707 (D.T.W.)

Supplementary information:

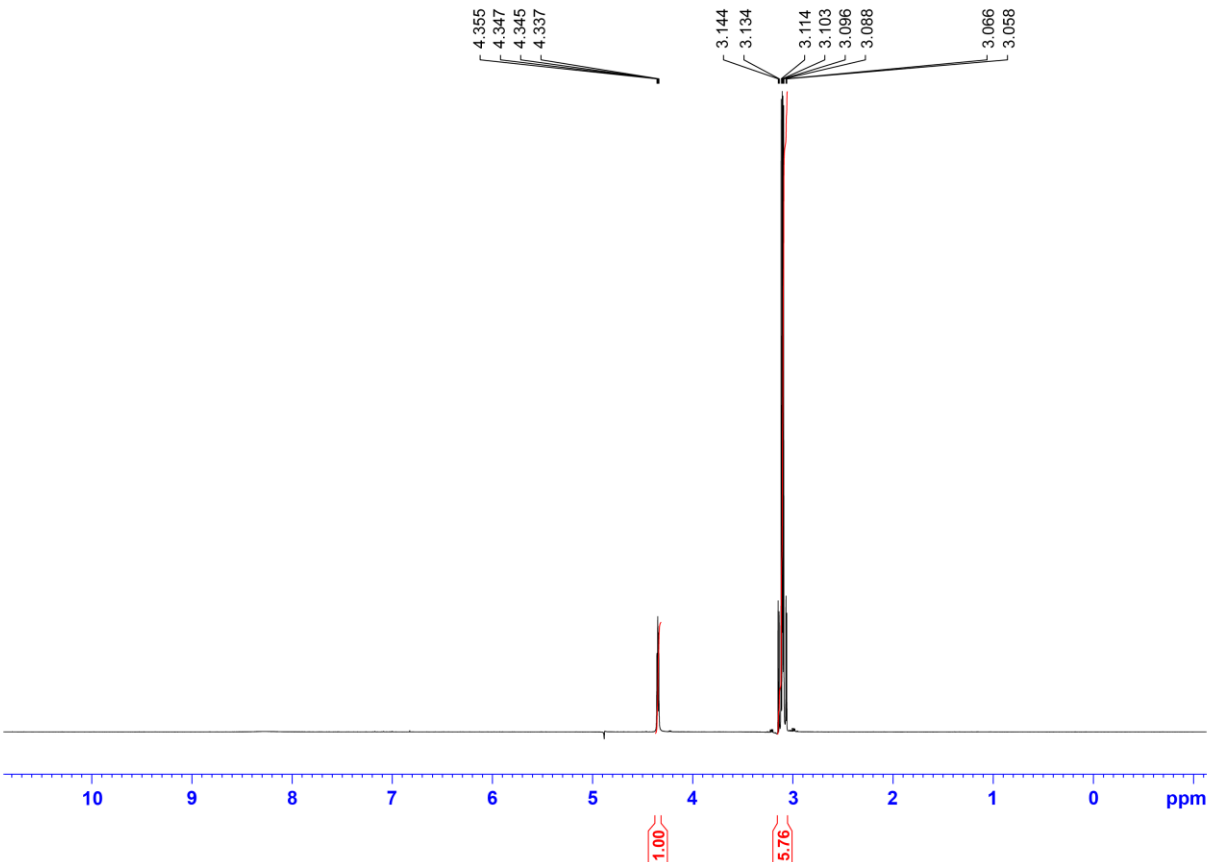

Figure S1: ^{1}H NMR spectrum of aspartic acid standard with integrals and chemical shifts. Sample dissolved in 90% H_2O/10% D_2O (*v*/*v*) (100 µM) and run at 290K on a Bruker Avance III 600 MHz spectrometer (Bruker, Billerica, MA, USA) equipped with a cryoprobe. Spectrum referenced to the water peak.

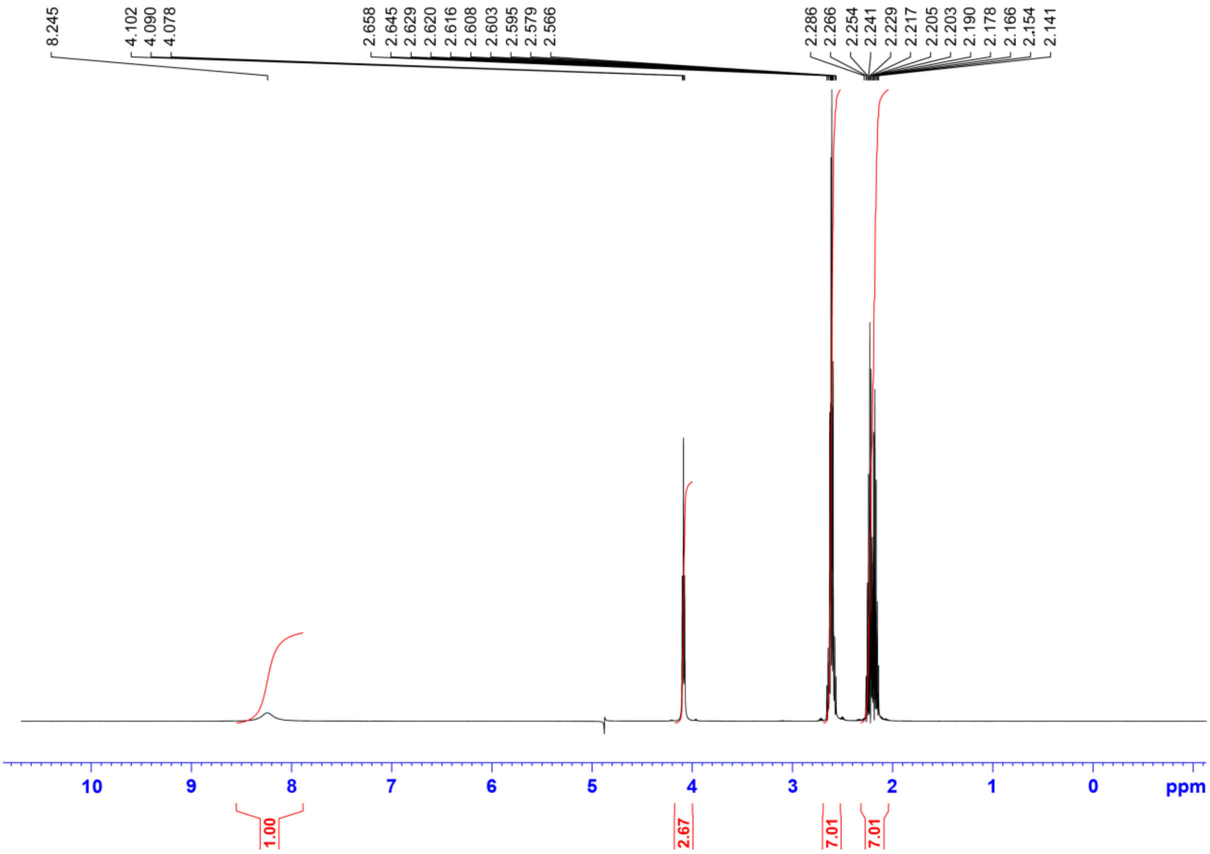

Figure S2: ^{1}H NMR spectrum of glutamic acid standard with integrals and chemical shifts. Sample dissolved in 90% H_2O/10% D_2O (*v*/*v*) (100 μM) and run at 290K on a Bruker Avance III 600 MHz spectrometer (Bruker, Billerica, MA, USA) equipped with a cryoprobe. Spectrum referenced to the water peak.

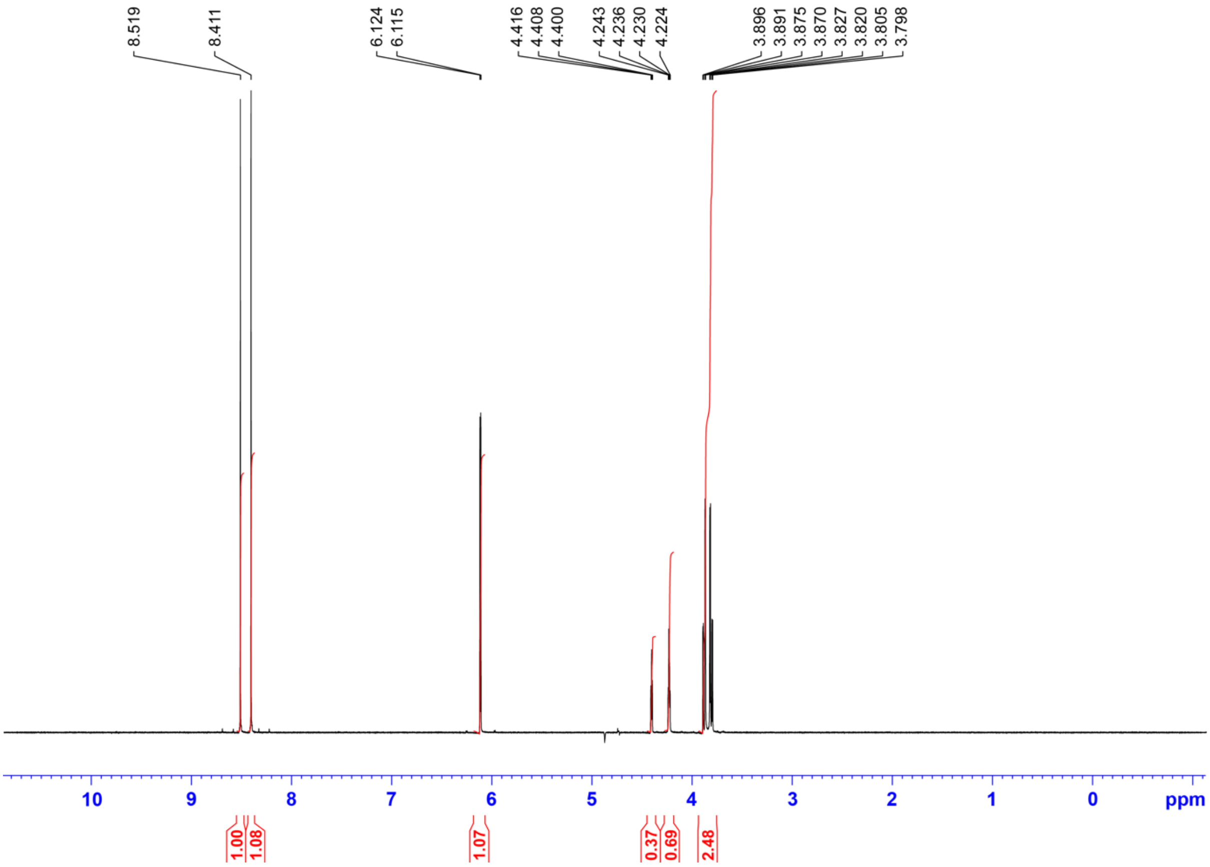

Figure S3: ^{1}H NMR spectrum of adenosine standard with integrals and chemical shifts. Sample dissolved in 90% H_2O/10% D_2O (*v*/*v*) (100 μM) and run at 290K on a Bruker Avance III 600 MHz spectrometer (Bruker, Billerica, MA, USA) equipped with a cryoprobe. Spectrum referenced to the water peak.

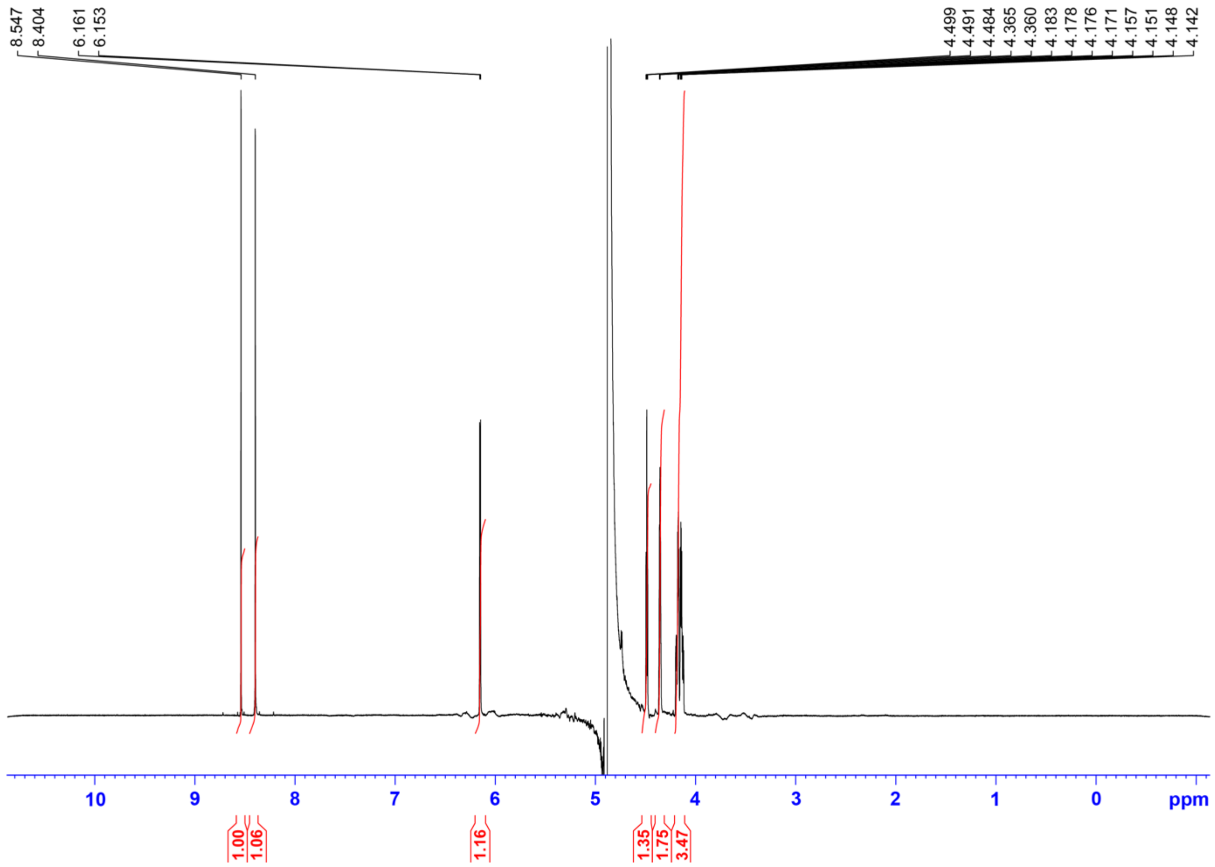

Figure S4: ^{1}H NMR spectrum of adenosine monophosphate standard with integrals and chemical shifts. Sample dissolved in 90% H_2O/10% D_2O (*v*/*v*) (100 μM) and run at 290K on a Bruker Avance III 600 MHz spectrometer (Bruker, Billerica, MA, USA) equipped with a cryoprobe. Spectrum referenced to the water peak. The calculated 90° pulse length for this sample was reduced and as a consequence full water suppression was not achieved.

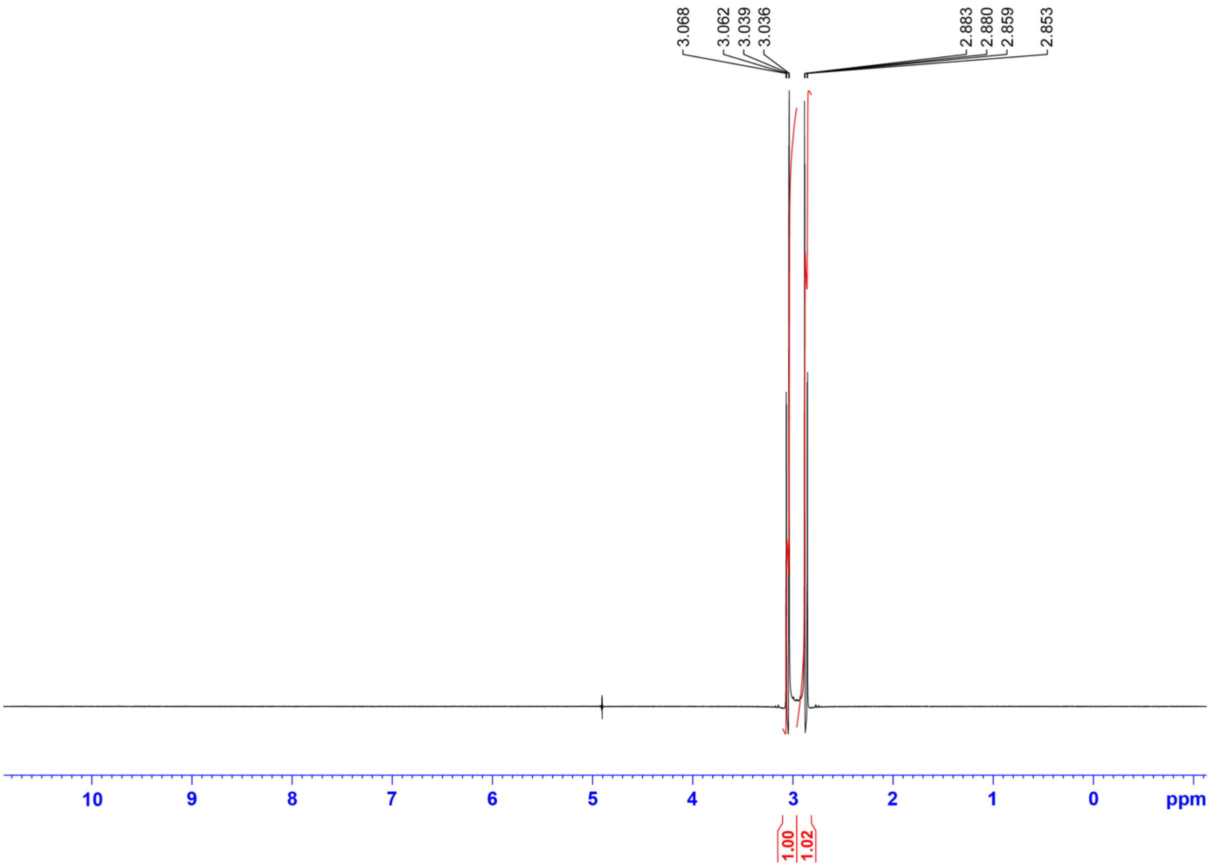

Figure S5: ^{1}H NMR spectrum of citric acid standard with integrals and chemical shifts. Sample dissolved in 90% H_2O/10% D_2O (*v*/*v*) (100 μM) and run at 290K on a Bruker Avance III 600 MHz spectrometer (Bruker, Billerica, MA, USA) equipped with a cryoprobe. Spectrum referenced to the water peak.

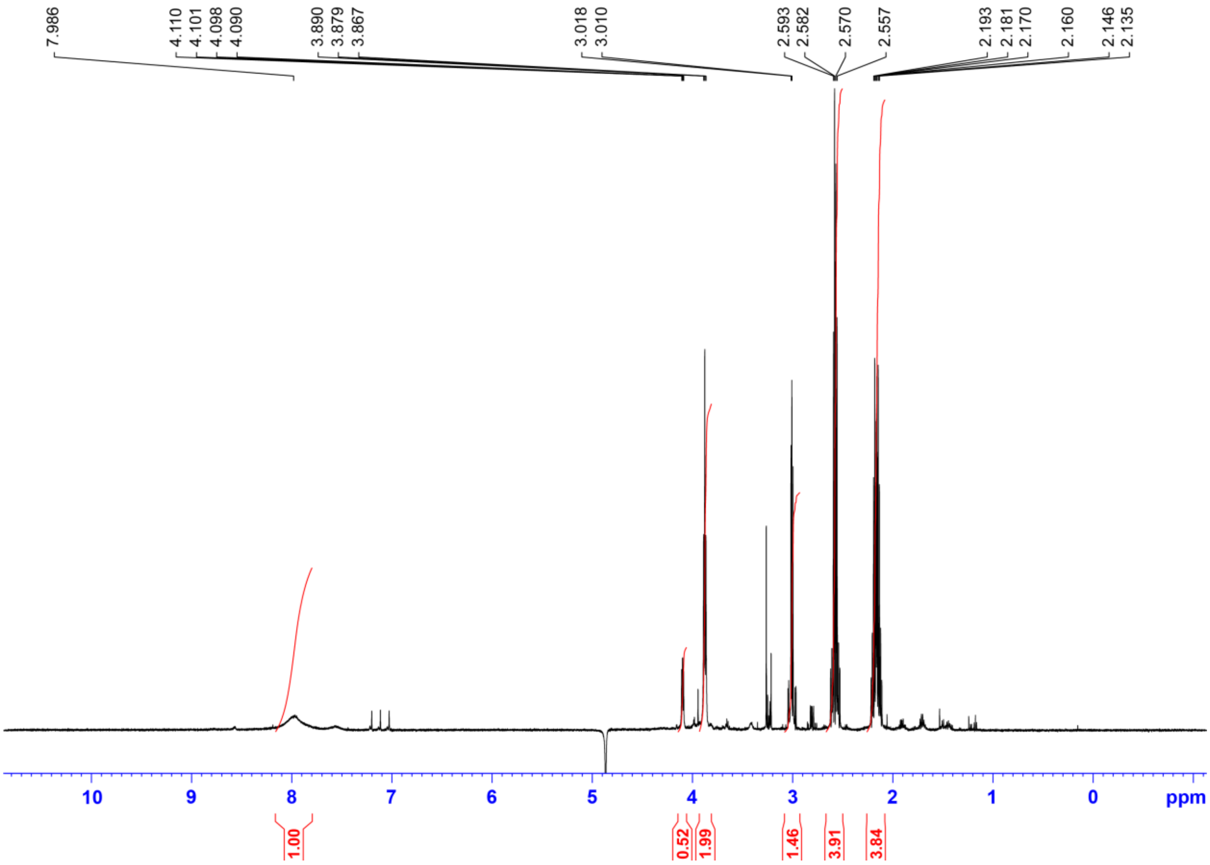

Figure S6: ^{1}H NMR spectrum of the RP-HPLC fraction of *H. waigiensis* containing aspartic acid and glutamic acid with integrals and chemical shifts. Sample dissolved in 90% H_2O/10% D_2O (*v*/*v*) (100 μM) and run at 290K on a Bruker Avance III 600 MHz spectrometer (Bruker, Billerica, MA, USA) equipped with a cryoprobe. Spectrum referenced to the water peak.

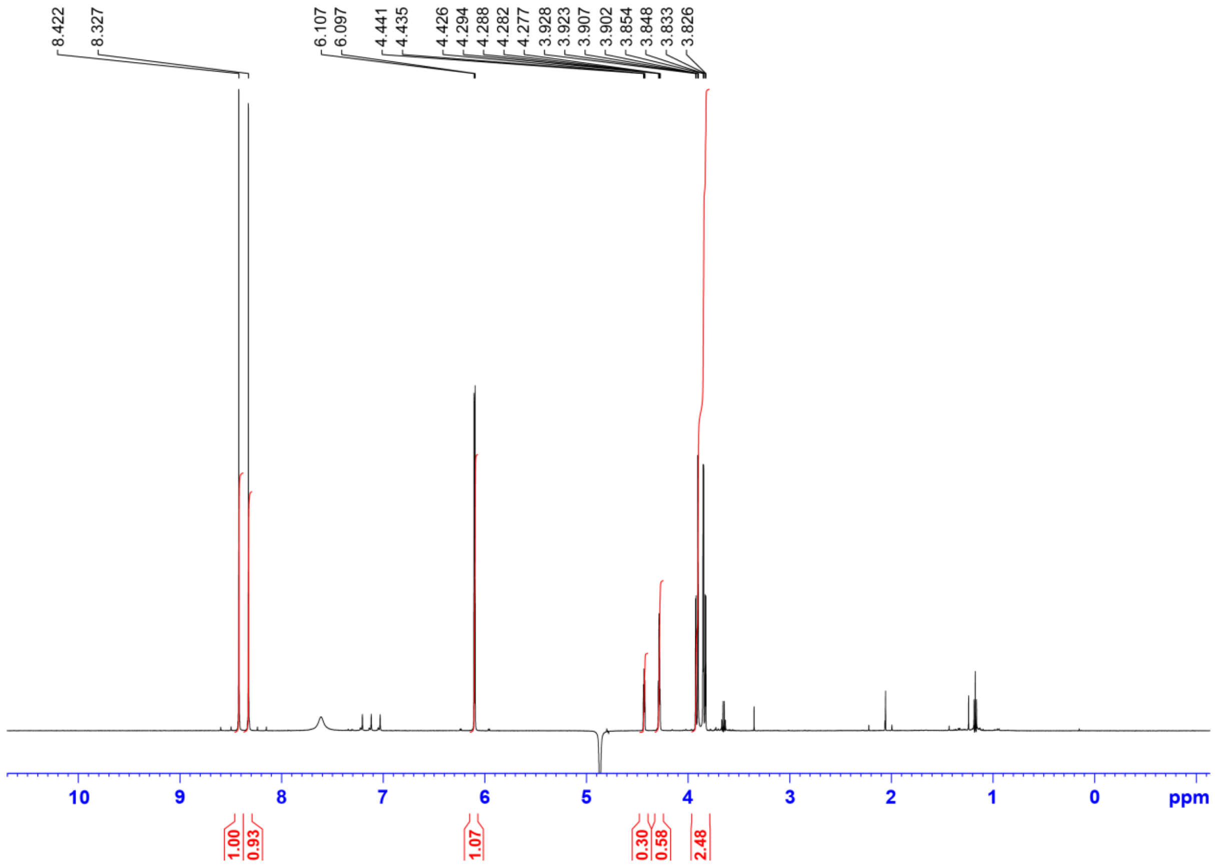

Figure S7: ^{1}H NMR spectrum of the RP-HPLC fraction of *H. waigiensis* containing adenosine with integrals and chemical shifts. Sample dissolved in 90% H_2O/10% D_2O (*v*/*v*) (100 μM) and run at 290K on a Bruker Avance III 600 MHz spectrometer (Bruker, Billerica, MA, USA) equipped with a cryoprobe. Spectrum referenced to the water peak. As evident from the adenosine monophosphate standard spectrum (Figure S4) where full water suppression was not achieved, the water suppression impacts the integrals of the adenosine peaks at 4.2 and 4.4 ppm.

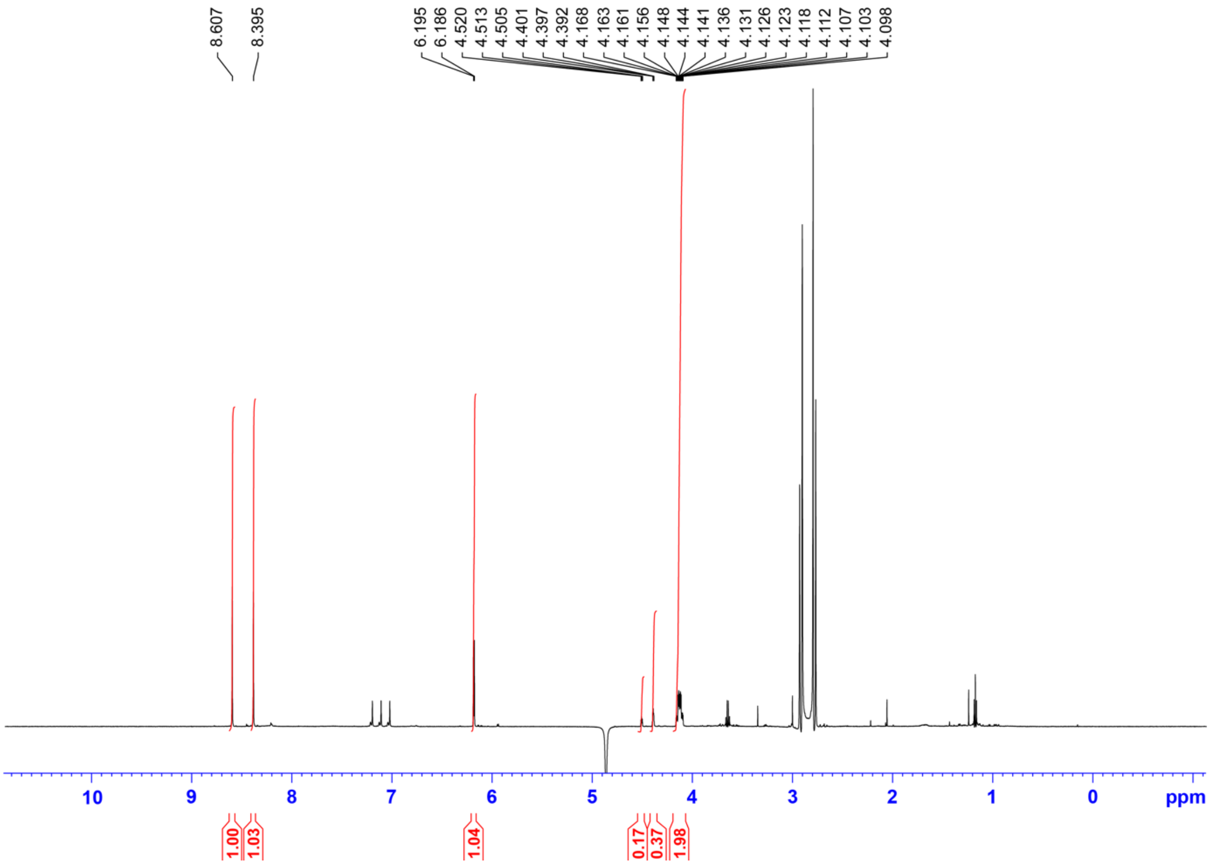

Figure S8: ^{1}H NMR spectrum of the RP-HPLC fraction of *H. waigiensis* containing adenosine monophosphate with integrals and chemical shifts. Sample dissolved in 90% H_2O/10% D_2O (*v*/*v*) (100 μM) and run at 290K on a Bruker Avance III 600 MHz spectrometer (Bruker, Billerica, MA, USA) equipped with a cryoprobe. Spectrum referenced to the water peak. Citric acid is also present just below 3 ppm. As evident from the adenosine monophosphate standard spectrum (Figure S4) where full water suppression was not achieved, the water suppression impacts the integrals of the adenosine monophosphate peaks at 4.2 and 4.5 ppm.

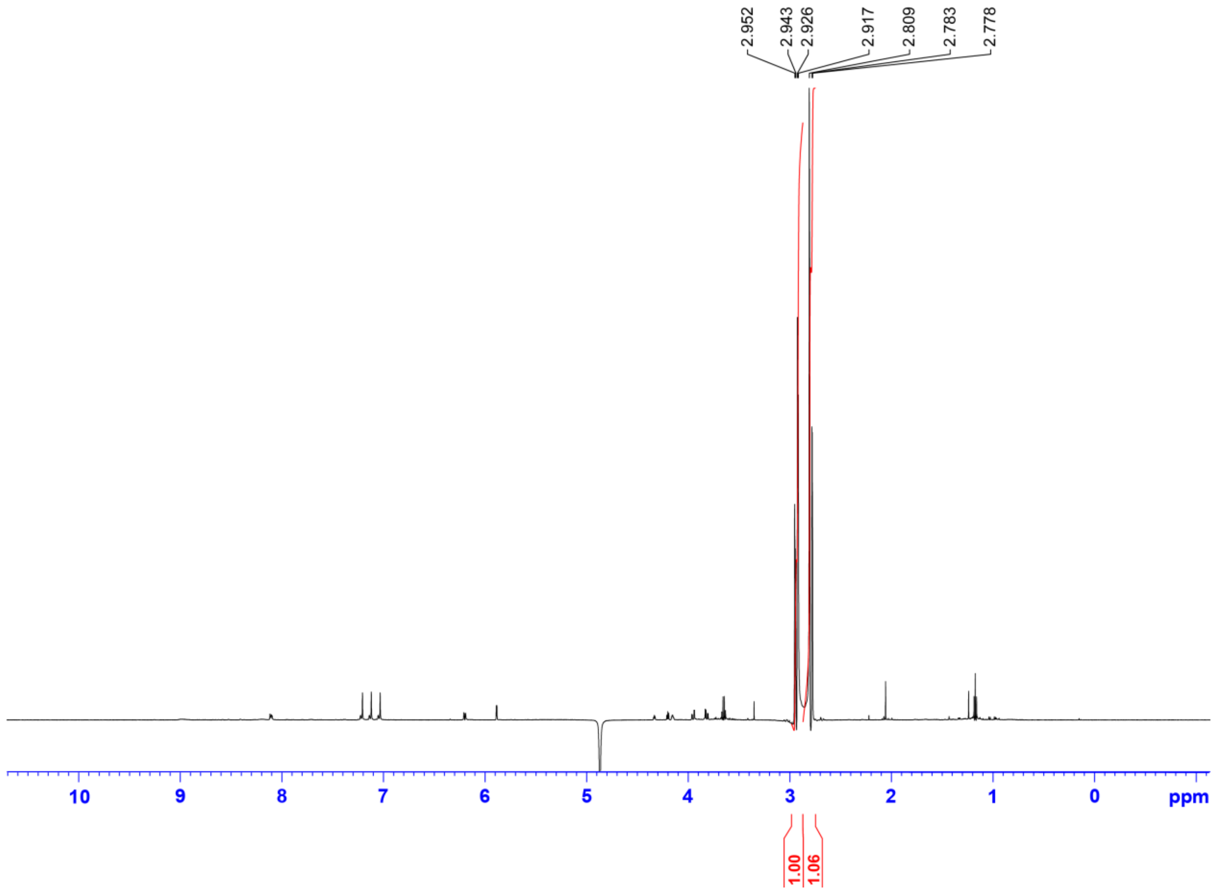

Figure S9: ^{1}H NMR spectrum of the RP-HPLC fraction of *H. waigiensis* containing citric acid with integrals and chemical shifts. Sample dissolved in 90% H_2O/10% D_2O (*v*/*v*) (100 μM) and run at 290K on a Bruker Avance III 600 MHz spectrometer (Bruker, Billerica, MA, USA) equipped with a cryoprobe. Spectrum referenced to the water peak.
